# Complete Genome Sequence of the 936-Type Lactococcal Bacteriophage *Caseus*JM1

**DOI:** 10.1128/genomeA.00059-13

**Published:** 2013-03-21

**Authors:** James Murphy, Jennifer Mahony, Douwe van Sinderen

**Affiliations:** a Department of Microbiology, University College Cork, Cork, Ireland; b Alimentary Pharmabiotic Centre, University College Cork, Cork, Ireland

## Abstract

The 936-type lytic bacteriophages are the most frequently encountered species infecting lactococcal dairy starters. Infection by members belonging to this species has a significant negative impact on the cheese production process. Here we report the complete genome sequence of the bacteriophage *Caseus*JM1, a 936-type phage isolated from an Irish dairy plant.

## GENOME ANNOUNCEMENT

Strains belonging to *Lactococcus lactis* represent important starter cultures in the dairy industry, and despite decades of research, such starter strains continue to suffer from phage attacks (1). Several strategies are employed to counteract the effects of phages, such as starter rotation, improved sterilization, and increased surveillance (2). However, several global studies have shown that 936-type phages remain one of the most frequently isolated species, sometimes leading to so-called slow or dead vats (3–5).

*Caseus*JM1 was isolated from a whey sample collected from a slow cheese vat in an Irish dairy plant. *Caseus*JM1 was identified as a 936-type phage based on transmission electron microscopy and multiplex PCR (6) (data not shown). *Caseus*JM1 was purified on a cesium chloride gradient, and 5 µg of genomic DNA was isolated as described previously (7).

The sequencing of the *Caseus*JM1 genome was conducted using a genome sequencer (GS) FLX titanium sequencer (Macrogen Inc., Seoul, South Korea). A 110-fold sequencing coverage was obtained using pyrosequencing technology on a 454 FLX instrument. The files generated by the 454 FLX instrument were assembled with GSassembler (454 Life Sciences, Branford, CT) to generate a consensus sequence. Several PCR products covering various sections of the genome were sequenced to ensure correct assembly and the resolution of any remaining base conflicts occurring within homopolymer tracts.

Open reading frames (ORFs) were automatically predicted using Heuristic Approach for Gene Prediction (8). Proteins with a minimum size of 30 amino acids were manually curated by determining the ribosomal binding site as well as the start and stop codons using the annotation software Artemis (http://www.sanger.ac.uk/resources/software/artemis/). Each of the determined ORFs was functionally annotated using BLASTp (9).

*Caseus*JM1 was found to have a double-stranded linear genome of 30,692 bp with a G+C content of 34.84%. A total of 51 ORFs were predicted and were divided into a modular organization based on previously published 936-type phages (4, 10) with early expressed (21 genes on the positive strand), late expressed (26 genes on the negative strand), and middle expressed (4 genes on the positive strand) genes. Of the ORFs identified, 34 were annotated as hypothetical, while those ORFs to which a function could be assigned (as based on blast searches) included genes specifying predicted structural proteins (major capsid protein, portal protein, tape measure protein, neck passage structure, receptor binding protein), DNA packaging proteins (small and large terminase subunit), and host lysis (holin and lysin), as well as a SAK kinase, and a single-stranded DNA binding protein. A putative single-stranded DNA-specific exonuclease was also found, an additional gene not frequently identified among the 936-type phages. Several predicted HNH endonucleases were also present in the genome. Comparative analysis revealed that *Caseus*JM1 exhibits a high level of homology to genomes of other 936-type phages (>85% identity). Continued analysis of the genomic organization and diversity is essential to help understand why these phages represent such a significant and constant threat to the dairy industry.

### Nucleotide sequence accession number. 

The complete genome sequence of lactococcal phage *Caseus*JM1 has been deposited in GenBank under accession number KC522412.
